# Signal amplification and quantification on lateral flow assays by laser excitation of plasmonic nanomaterials

**DOI:** 10.7150/thno.44298

**Published:** 2020-03-15

**Authors:** Haihang Ye, Yaning Liu, Li Zhan, Yilin Liu, Zhenpeng Qin

**Affiliations:** 1 Department of Mechanical Engineering, University of Texas at Dallas, Richardson, Texas 75080, USA.; 2 Department of Mechanical Engineering, University of Minnesota, 111 Church Street SE, Minneapolis, Minnesota 55455, USA; 3 Department of Bioengineering, University of Texas at Dallas, Richardson, Texas 75080, USA; 4 Center for Advanced Pain Studies, University of Texas at Dallas, Richardson, Texas 75080, USA; 5 Department of Surgery, The University of Texas Southwestern Medical Center, 5323 Harry Lines Blvd, Dallas, Texas 75390, USA

**Keywords:** lateral flow assay, gold nanoparticles, SERS, signal amplification and quantification, nanoparticle heating

## Abstract

Lateral flow assay (LFA) has become one of the most widely used point-of-care diagnostic methods due to its simplicity and low cost. While easy to use, LFA suffers from its low sensitivity and poor quantification, which largely limits its applications for early disease diagnosis and requires further testing to eliminate false-negative results. Over the past decade, signal enhancement strategies that took advantage of the laser excitation of plasmonic nanomaterials have pushed down the detection limit and enabled quantification of analytes. Significantly, these methods amplify the signal based on the current LFA design without modification. This review highlights these strategies of signal enhancement for LFA including surface enhanced Raman scattering (SERS), photothermal and photoacoustic methods. Perspectives on the rational design of the reader systems are provided. Future translation of the research toward clinical applications is also discussed.

## Introduction

**Figure 1:**
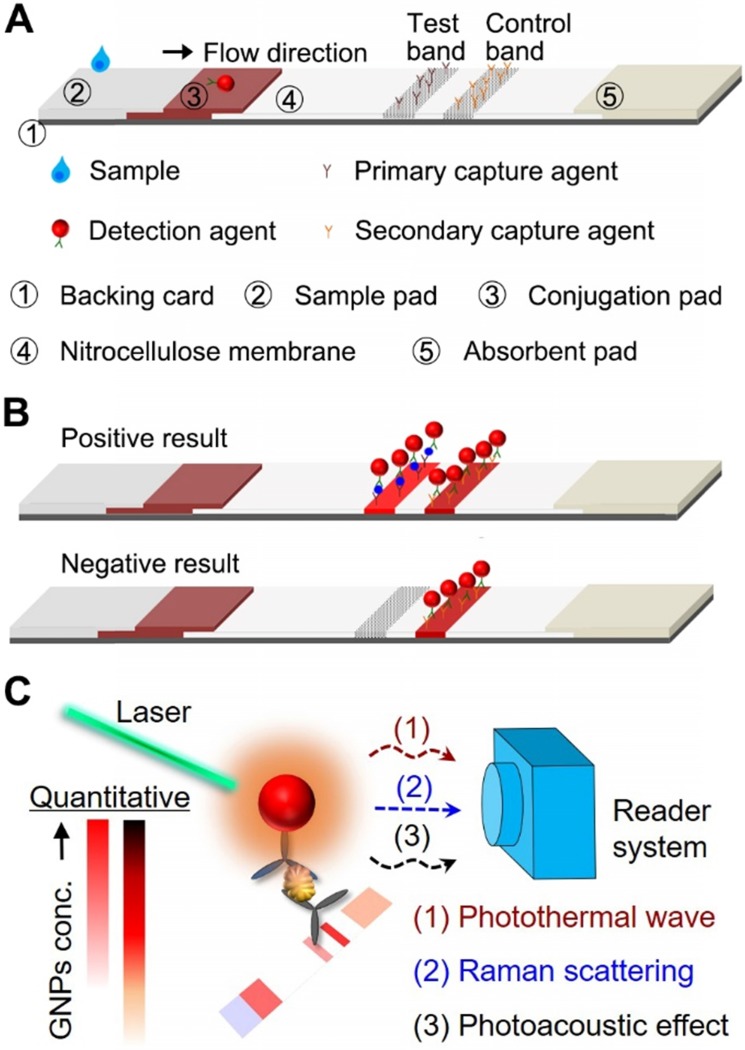
Schematics showing the (A) working principle and components of a sandwich-type LFA. (B) Signal readout for positive and negative results of LFA, where the test band shows the signal of detection and the control band functions for the validation. (C) The outline of sensing modes induced by the laser-GNP interaction for the sensitive and quantitative detection on LFA that were elaborated in the review.

Lateral flow assay (LFA) is one of the most widely used point-of-care (POC) diagnostic tests and can be performed simply and rapidly without specially trained personnel 1-3. LFA is also cost-effective that allows the easy access to large populations in resource-limited settings. A standard LFA is performed on a paper-based device, and its architecture consists of a sample pad, a conjugation pad, a capillary membrane, and an absorbent pad on a plastic backing card (Figure 1A). For a sandwich-type LFA, the presence of both test and control bands indicates a positive result (Figure 1B). Alternatively, competitive-type LFA is developed with opposite sensing mode. Details of this LFA format could be found in published work elsewhere 4.

Broader applications of LFA have been mainly limited by two factors: the lack of quantification and the low sensitivity, due to the weak optical signal from the detection agents 5. In fact, the conventional LFA is intentionally designed for qualitative (i.e., yes or no) or semi-quantitative (refers to a color chart) tests so that the results could be read directly by naked eyes without additional instruments 6. Thus, it only works for the tasks that involve a high concentration of analytes (e.g., ng/mL). These intrinsic limitations prevent the conventional LFA from becoming modern POC diagnostics. For example, the early-stage detection and management of severe and infectious diseases (e.g., COVID-19, 7) requires rapid and sensitive detection at low analyte concentrations. So far, the analytical performance of conventional LFA is often insufficient to be standalone tests.

In the past decades, extensive efforts have been devoted to improving the sensitivity and quantification for LFA. Considering that the detection agent is the key for the signal generation, one idea is to utilize them with stronger signals to improve the diagnostic performance of LFA 8, 9. Therefore, different types of detection agents have been developed including organic dyes 10, quantum dots 11, 12, upconverting nanoparticles (NPs) 13, latex beads 14, 15, noble-metal NPs 16, 17, carbon NPs 18, 19, colloidal selenium 20, and magnetic NPs 21. Notably, these material innovations have advanced the limit of detection (LOD) into the level of pg/mL (vs. ng/mL of conventional LFA) and significantly broadened the applications. High-quality and scalable manufacturing of these nanomaterials is required to translate these techniques from the laboratory experiments to clinical use. Other strategies using additional reagents like metallic enhancement 22, 23, enzyme-based catalytic reactions 24, 25, and a combination of them 26 also lead to remarkable LOD improvement but add complexity to the LFA operation.

An attractive strategy that amplifies signals based on the current LFA design has emerged in the last 5-10 years. The strategy mainly relies on the laser excitation of plasmonic gold NPs (GNPs) and the subsequently generated signal is much stronger than the color signal, resulting in the LOD enhancement. Note, GNPs are the most frequently used detection agents for *in vitro* diagnostics, especially in LFA. Like other plasmonic nanomaterials, GNPs feature a unique phenomenon called localized surface plasmon resonance (LSPR), making themselves ideal candidates in this revolution 27, 28. The LSPR comes from the strong interaction between light and GNPs, where the surface electrons of GNPs oscillate collectively with the electric field of light 29. It also leads to localized effects including enhanced electromagnetic field and eventually heating 30-32. Taken together, applying laser on the GNPs-based LFA provides a new route to benefit its analytical performance without modifying the current architecture and operation. There are several advantages to this approach. Firstly, it keeps the simplicity and rapidness of LFA and doesn't add complexity in performing the assay. Secondly, it offers additional sensing modes to the conventional LFA. The as-generated signals from GNPs can also serve as indicators to determine target concentration. Thirdly, a more sensitive and quantitative detection could be achieved by a laser-reader system. Reading the color signal by naked eyes, especially for weak color change, inevitably causes inconsistent result in LFA 2. With the assistance of a reader, robust and consistent results could be realized. Lastly, the laser-reader systems could potentially be miniaturized as handheld devices with all-in-one platform, which is highly desirable for POC diagnostics. To our knowledge, a review of this special topic on laser-induced signal amplification for GNPs-based LFA is not available and will provide intriguing insights from both scientific findings and clinical applications.

In this review, we present the current status of LFA enhancement strategies that emerged over the past decade. Specifically, we will focus on methods that take advantage of laser excitation of plasmonic NPs and directly enhance signal based on the current LFA architecture, including surface enhanced Raman scattering, photothermal and photoacoustic methods (Figure 1C). For each method, we will introduce the principle of signal enhancement strategies, show its implementation on LFA and impacts on the sensitivity and quantification, examine the design of reader systems, and discuss potential limitations. We anticipate that the signal amplification techniques, as well as the perspectives provided in this article, serve as a guideline to explore novel sensing modes and detection approaches for the development of LFA towards highly sensitive POC diagnostics.

## Laser-induced Signal Amplification Techniques

### Surface enhanced Raman scattering (SERS) enhanced signal

#### Principle

Surface enhanced Raman scattering (SERS) is a sensing technique that generates and amplifies the inelastic light scattering of molecules when they are adsorbed on the metals (e.g., gold, silver, and copper) 33. This scattering signal could be enhanced (up to 10 orders of magnitude) by modulating the frequency of excitation light and LSPR of the metallic nanomaterials, relevant to the Stokes and/or anti-Stokes lines of molecules 34. In SERS-based assay, specific tags made of nanostructures and molecules with known Raman fingerprints are the detection agents. Measuring the peak intensity of Raman molecules allows for the quantification of particle and analyte concentrations. Consequently, SERS has become a powerful tool in diagnostics due to its rapid, sensitive, and multiplexed outcomes 33-35.

#### LFA demonstration

**Figure 2:**
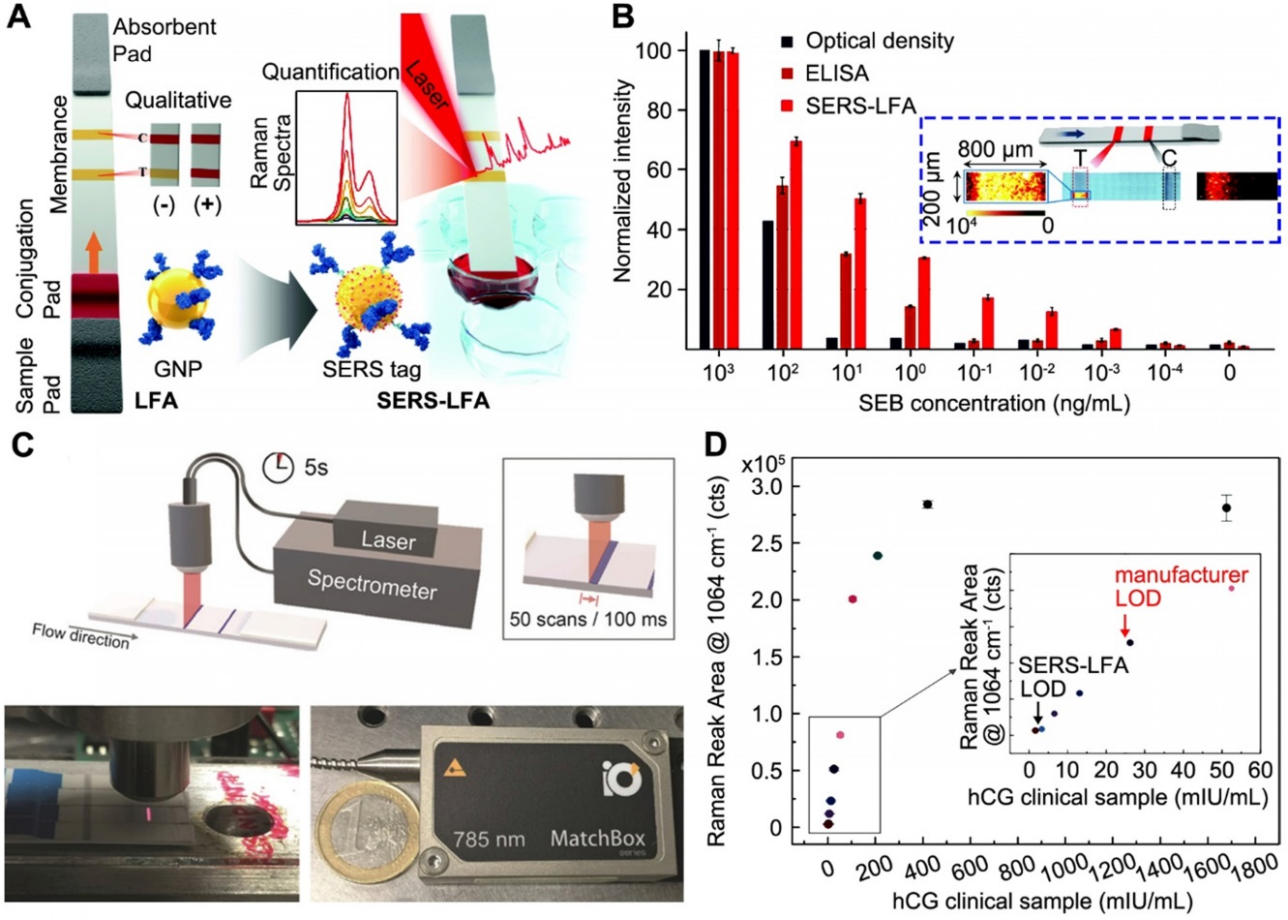
Surface-enhanced Raman scattering (SERS)-based LFA with enhanced detection sensitivity. (A) Schematics showing the principle of measuring SERS signal on LFA strips with GNPs-based SERS tags. (B) Comparison of the analytical results obtained from the optical density of conventional LFA strips, ELISA, and present SERS-LFA strips in detection of staphylococcal enterotoxin B (SEB). Inset in blue box shows a typical focused scanning of test band (200 μm x 800 μm) and whole control band of LFA strip by SERS spectroscope system. T and C stands for test band and control band, respectively. (C) Schematic representation of a portable SERS reader with line-focused optical fiber probe laser. Scanning steps and time is shown in the box. Photographs show the custom-designed optical fiber probe and a 785 nm diode laser. (D) Dose response curve of the SERS signal after applying different concentrations of hCG clinical samples. Inset shows a linear SERS response at low hCG concentrations and the vertical line marks the LOD of SERS-LFA and commercially available LFA kits. Adapted with permission from 37, 54, copyright 2016 The Royal Society of Chemistry and 2019 The Authors, published by Wiley-VCH Verlag GmbH & Co. KGaA., respectively.

Utilizing SERS for LFA has been explored as a highly sensitive assay platform since 2007 36. In SERS-LFA, the assay operation is identical to that of a conventional LFA, except for the tags' preparation. In the work by Hwang et al. 37, Raman molecules and antibody-coated hollow GNPs were utilized as SERS tags for the LFA (Figure 2A). For the SERS signal readout, a completed LFA strip was scanned in a standard Raman microscope system for the measurement, where the SERS peak intensity of test band was monitored for quantitative analysis. Using staphylococcal enterotoxin B (SEB) as a model, the authors performed SERS-LFA, conventional LFA, and ELISA for comparison. SEB is one of the bacterial superantigens secreted from Staphylococcus aureus that infects immune system profoundly and leads to a wide range of infectious diseases 38. The result shown in Figure 2B demonstrates that SERS increased detection range and improved LOD of LFA by 10^4^-fold, and it was even 10^3^-fold lower than that of ELISA. Particularly, for each strip characterization, a focused area (200 μm × 800 μm) of test band was used to collect the SERS signal, while the control band was tested for verification (see the inset in Figure 2B). Also, due to the uneven distribution of SERS signal on the test band, the average intensity of scanned regions was further used for the quantitative analysis. The concept of combining SERS with LFA for nucleic acids detection was also reported by the same group 39, and soon after that, this assay platform has been widely extended to different targets, including disease biomarkers 40 and pathogens 41.

One major factor leads to highly sensitive SERS-LFA is deploying NPs with stronger plasmonic coupling effect. For example, nanostructures with “hot spots” were reported to enhance SERS signals, including plasmonic NPs with rough surface, coupled NPs with nanogaps, and multibranched nanostructures 42-44. Also, the specific interaction (e.g., electron transfer) between the Raman molecules and the NPs allows further amplification of SERS signal 34. Meanwhile, the stability of Raman signals is another direction that requires carefully design of the SERS tags. For instance, NPs with high uniformity and protection layers enable evenly distributed signal and avoid the loss of Raman reporters, respectively 45, 46. Therefore, optimizing the shape and composition of the plasmonic NPs has great promise in the development of SERS-based diagnostics for the amplified and robust signal readouts.

SERS can also lead to multiplexed detection on LFA. Specifically, SERS tags encoded with different Raman molecules are conjugated to the detection antibodies for multiple targets 47, 48. For example, Doering et al. demonstrated for the first time using Nanoplex Biotags (Au@SiO_2_ NPs, commercially available from Oxonica, Inc.) for the multiplexed detection of Flu A, Flu B, and respiratory syncytial virus on a standard LFA 36. However, combining multiple targets in one test band, the as-obtained SERS spectrum could show overlapped or close peaks and may impact on the sensitivity of LFA 49, 50. Arranging parallel test bands on the membrane could also achieve multiplexed detection, where each band associates to identify one of the targets 51. This inevitably increases the assay time and reduces the signal throughput 52. In addition, construction of microarray on LFA strips offers a promising solution to shorten the sample-to-answer time. For example, Zhang et al. demonstrated a multiplexed detection of respiratory tract infection pathogens (up to 11) by grouping two SERS tags and a 2×3 microarray as test dots on the LFA 53. Collectively, further work could put more efforts in the nanostructure design, strip construction, and Raman reporter's selection for multiplexed detection on SERS-LFA.

#### Reader development

As a proof-of-principle, bulky Raman microscope system was used to measure the SERS signal of LFA, yet impractical for POC deployment. Recently, Tran et al. reported a portable SERS reader designed for rapid scanning of the LFA strips 54. The compact setup mainly composed of an optical fiber probe and a 785 nm diode laser (Figure 2C). Taking advantage of the line focus of optical fiber probe, the laser illumination covers the entire width of strip and moves across step by step (0.1 s for each step and 50 steps per band), achieving 5 s acquisition time for the test band (4 mm × 0.8 mm) within an individual scan. In addition, this compact SERS-LFA platform showed improved diagnostic performance for the human chorionic gonadotropin (hCG) detection. Notably, in the work by Tran et al., Au-Ag core-satellite NPs were used as detection agents, which provided 4-fold visual LOD enhancement over the commercial LFA kits. While for SERS detection, the reader further lowered the LOD by 4-fold, thereby 16-fold improvement in total over the commercial LFA kit (Figure 2D).

#### Limitations

There are several limitations of SERS-LFA technique. Firstly, special design of the SERS tags with strongly enhanced local electromagnetic field are required to generate sufficient and robust Raman signals. For example, Au@SiO_2_ core-shell NPs, hollow GNPs, Au-Ag core-satellite NPs have been used in the above-mentioned work 36, 37, 54. This could potentially lead to complexity and difficulty in SERS molecule encoding, antibody conjugation, and maintaining shelf stability. Secondly, the trade-off occurs between signal intensity and data acquisition settings. For instance, point scanning with long acquisition time favors for strong Raman signal. However, this compromises the working efficiency and only samples a limited fraction of the test band, which could lead to under-sampling and inconsistent results from test to test (i.e., low reproducibility). This issue also exists in some commercially available SERS readers (e.g., Snowy Range 'CBEx' Raman spectrometer, Laramie, USA) 55. In contrast, increasing sampling area and reducing acquisition time result in significantly inferior performance. For instance, Hwang et al. reported 10^4^-fold improvement by point scanning part of the test band (200 μm × 800 μm) with long acquisition time (6 min), while Tran et al. reported 4-fold improvement by line scanning the whole test band (4 mm × 0.8 mm) with short acquisition time (5 s). In addition, the line scanning requires more powerful laser (120 mW) compared with the point-scanning Raman microscope system (3 mW).

### Photothermal enhanced signal

#### Thermal contrast amplification (TCA)

##### Principle

The heat generation of GNPs has been widely studied in the area of photothermal therapy for cancer treatment 56-59. Briefly, when the LSPR of GNPs matches with the laser wavelength, they can efficiently convert light energy (photons) into heat. The amount of heat generation could be estimated by the following equation:

*Q*= N*Q*_nano_= N*C*_abs_*I*

where the total heat generation (*Q*, W/m^3^) is the accumulated contribution of individual GNP (*Q*_nano_, W) and the particle concentration of GNPs (N, number/m^3^). *Q*_nano_ could be further written as the product of GNP absorption cross-section (*C*_abs_, m^2^) and laser intensity (*I*, W/m^2^). Clearly, the heat is proportional to the number of GNPs and laser energy applied within the detection region, and the *C*_abs_ is associated with the physical parameters of GNPs (e.g., size and shape) 32. This sets the basis to use the heat generation from GNPs and integrate thermography techniques for potential diagnostic applications.

##### LFA demonstration

**Figure 3:**
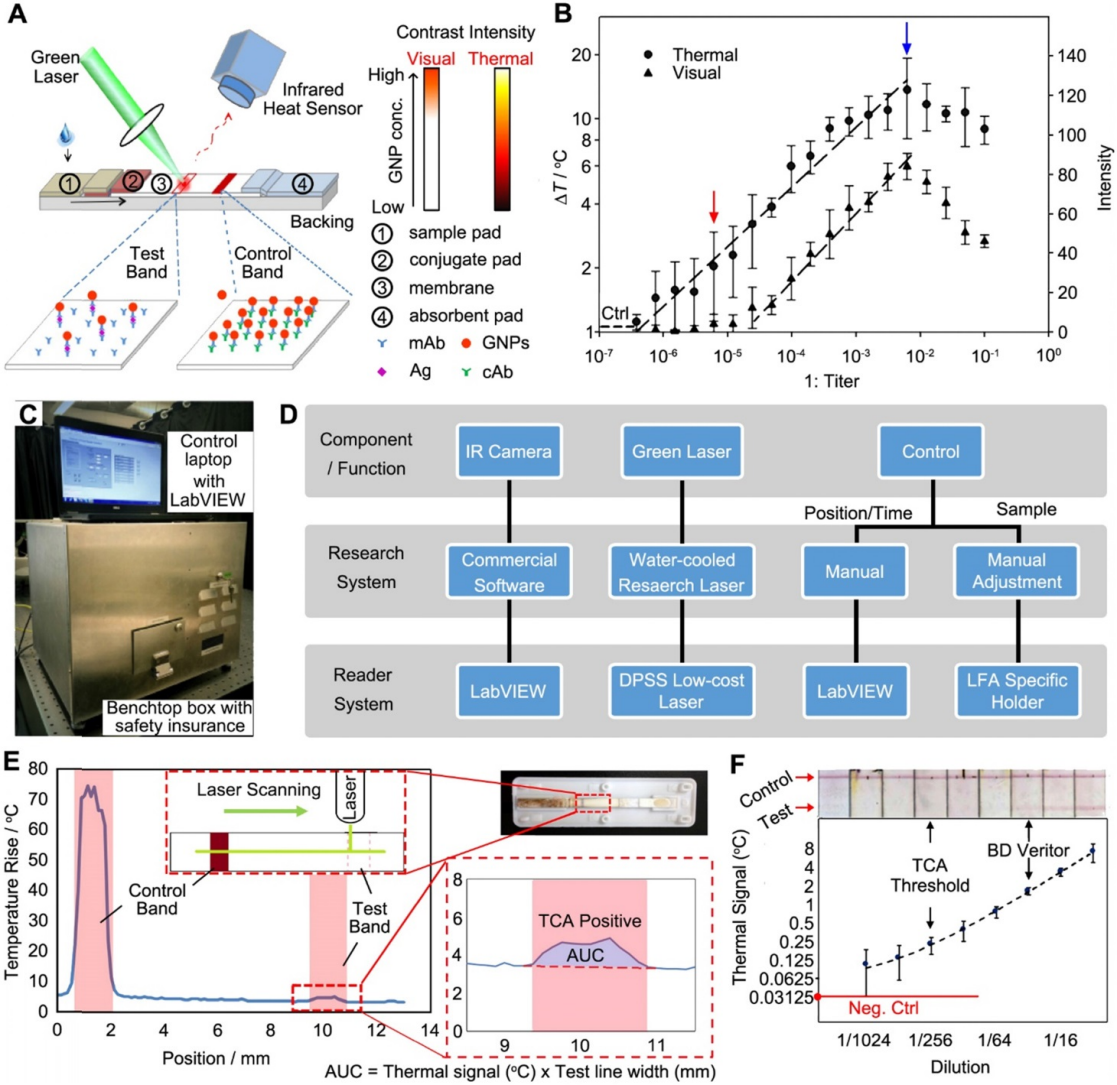
Thermal contrast amplification (TCA) technique for LFA. (A) Schematics showing the detection principle of TCA technique working on LFA strip. (B) Dose response curves of the TCA and colorimetric signals of LFA for CrAg. The “Ctrl” means the background signal from control sample (water). (C) A photograph of the benchtop reader system. (D) Comparison of a TCA research system and the reader system shown in (C). (E) TCA reader algorithm for detection and quantification of temperature rise in an LFA strip. The area under the curve (AUC) analysis was performed along the strip covering the control and test bands for the signal acquisition. The result was obtained from a visual-negative malaria First Response LFA kit as shown in inset. (F) Quantitative results of representative LFA strips using the TCA benchtop device against visual images. Samples are different dilutions of influenza A positive swabs extraction. LFAs are from BD Veritor. Adapted with permission from 61, 67, copyright 2012 John Wiley and Sons and 2016 American Chemical Society, respectively.

Qin et al. demonstrated for the first time using thermal response of GNPs to improve the LFA 61. Essentially, a completed LFA strip was irradiated with a 532 nm laser (power = 0.01 W) to activate the GNPs and the thermal contrast signal was recorded by an IR camera (Figure 3A). The thermal analysis of test band gave quantitative results as a function of the GNP concentrations. The analytical performance of FDA-approved LFA strips for detecting cryptococcal antigen (CrAg) was evaluated through the thermal contrast signal. CrAg testing is urgently required for cryptococcal meningitis diagnosis, especially in immunocompromised adults 62. Figure 3B shows the TCA produced a 32-fold enhancement in the detection sensitivity and extended detection dynamic range over the visual detection. Note the blue arrow marks the high dose “hook” effect with decreased visual and thermal intensity, which can be addressed by diluting the target sample or engineering the format of strip 6. The TCA technique was further validated in clinical cerebral spinal fluid samples with known CrAg titers from patients with and without cryptococcal meningitis. Recently, clinical cohort study was performed using the commercial CrAg LFA kits (Immy, Inc., USA) assisted with TCA technique 63.

The shape and size of the GNPs played an important role in improving their photothermal property and thereby the performance of diagnostics. Changing the spherical GNPs into gold nanorods (GNRs) or gold nanoshells (GNSs) could largely increase the *C*_abs_ per particle volume 64. As a result, under same condition (i.e., particle concentration and laser power), GNRs produce about 10-fold more heat than the spherical GNPs and GNSs when normalized against the particle volume. Moreover, when combined with low-absorbing substrates as the backing materials (e.g., glass and plastic) and higher laser power (i.e., 0.01 W to 1 W), GNRs-based TCA-LFA could have the potential to achieve 10^4^-fold improvement over the visual LOD of LFA 61. In addition, Hu et al. reported employing gold nanocages with superior photothermal conversion efficiency as detection agents for TCA-LFA, where a 6-fold enhancement of the LOD has been achieved compared with the visual LFA 65. Zhan et al. also studied the size impact of GNPs on the sensitivity of LFA 66. Importantly, the size of GNPs not only affects their optical and thermal properties, but also influences the migration speed and antibody binding events within the membrane. By modelling these key parameters (i.e., size and concentration of GNPs, migration rates, binding reaction, and diffusion), Zhan et al. found that larger GNPs could improve the assay performance due to more antibodies on the surface (favoring the binding reaction) and size-dependent properties (larger size resulting in stronger signal generation). For example, 100 nm GNPs yield a 256-fold sensitivity enhancement under the TCA detection mode compared to the colorimetric signal of 30 nm GNP-based LFA. However, even larger GNPs (e.g., 400 nm) would have difficulties in migrating across the strip due to the slower diffusion rates. This inevitably decreases GNP capture and thus the LFA sensitivity. Similar investigation of size impact on the LOD of LFA has also been reported by Loynachan et al. 17.

##### Reader development

Development of benchtop and portable devices is critical to further translate the technology. The initial detection system reported in 61 is only available in a research lab (i.e., a large laser system on an optical table) with manual control and a simple reading algorithm. In a follow-up study, the same group developed a benchtop TCA reader prototype (46 cm × 34 cm × 34 cm size, 11.7 kg weight, Figure 3C) that is low-cost and compact with automatic data acquisition and analysis 67. The overall design of the laser-reader system was shown in Figure 3D. In the benchtop device, a continuous wave (CW) laser was employed, of which the cost was significantly reduced but the performance was comparable to the original research laser (Spectra-Physics, Millennia Vs) under the same output power. Other improvements over the research system include automation of a stepper motor and an optical shutter through LabVIEW, as well as the LFA-holding cassettes that could align LFA strips of different sizes and geometries under the laser for reproducible reading. However, the IR camera with software for signal reading and data analysis wasn't changed due to the requirement of spatial resolution and user interface. Based on this benchtop device, a two-stage algorithm was used (Figure 3E): 1) the temperature rise was obtained by capturing multiple points (vs. single point in previous work 61) of test band subtracting the background temperature rise (outside test band); 2) area under curve (AUC) analysis of the TCA signal pattern was performed for the data reduction and quantitative results. The development of a handheld and portable device for TCA was conceptualized and initial assessment on the individual reader components such as a portable laser and a cheaper IR sensor was performed. Further work has been undergoing to build and test a POC device based on the TCA technique.

To validate the analytical performance of benchtop device, commercially available LFA kits for three diseases (influenza A, malaria, and C. difficile) were used. Both qualitative (i.e., subjective evaluation of TCA data) and quantitative (i.e., AUC analysis) results could be obtained (for simplicity, only influenza A kits were shown in Figure 3F). The TCA reader showed 8-fold enhancement over the visual recognition for all cases. For the quantitative analysis, a prominent linear trend was fitted, indicating the correlation of signal to the target concentration in the sample. In addition, the benchtop TCA reader was carried out to perform test on clinical samples, demonstrating a 4- to 8-fold improvement of analytical sensitivity. Importantly, TCA reader is a simple add-on reader for commercial LFAs without any additional modification.

##### Limitations

Although there are numerous benefits of TCA reader, some limitations need to be addressed before rigorous field tests. One is the reading time for each test. Currently, 10-20 min are required for data acquisition and analysis, which should ideally be shortened to less than 3 min per test. Also, due to the random distribution of GNPs in test band and point- scanning of the TCA method, reproducible measurements must be carefully considered. Therefore, it is important to develop a portable reader with robust and consistent results since the precise temperature measurements are used as the “thermal” signal.

#### Thermophotonic lock-in imaging (TPLI)

##### Principle

Thermophotonic lock-in imaging (TPLI) is a thermography testing technique for industrial evaluation of materials and devices 68. In this technique, a low-power, continuous, and intensity- modulated laser is introduced to produce a controlled thermal wave inside the feature of interest. The lock-in demodulation evaluates the detected signal that carries information of sample inhomogeneities using the excitation light as reference 69. In other words, the absorbers located at different depths inside the feature possess different optical scattering and absorption coefficients, contributing to the depth- integrated IR signal. Recent studies have successfully demonstrated the TPLI application to the biological samples such as early detection of dental caries in human teeth 69, 70.

##### LFA demonstration

**Figure 4:**
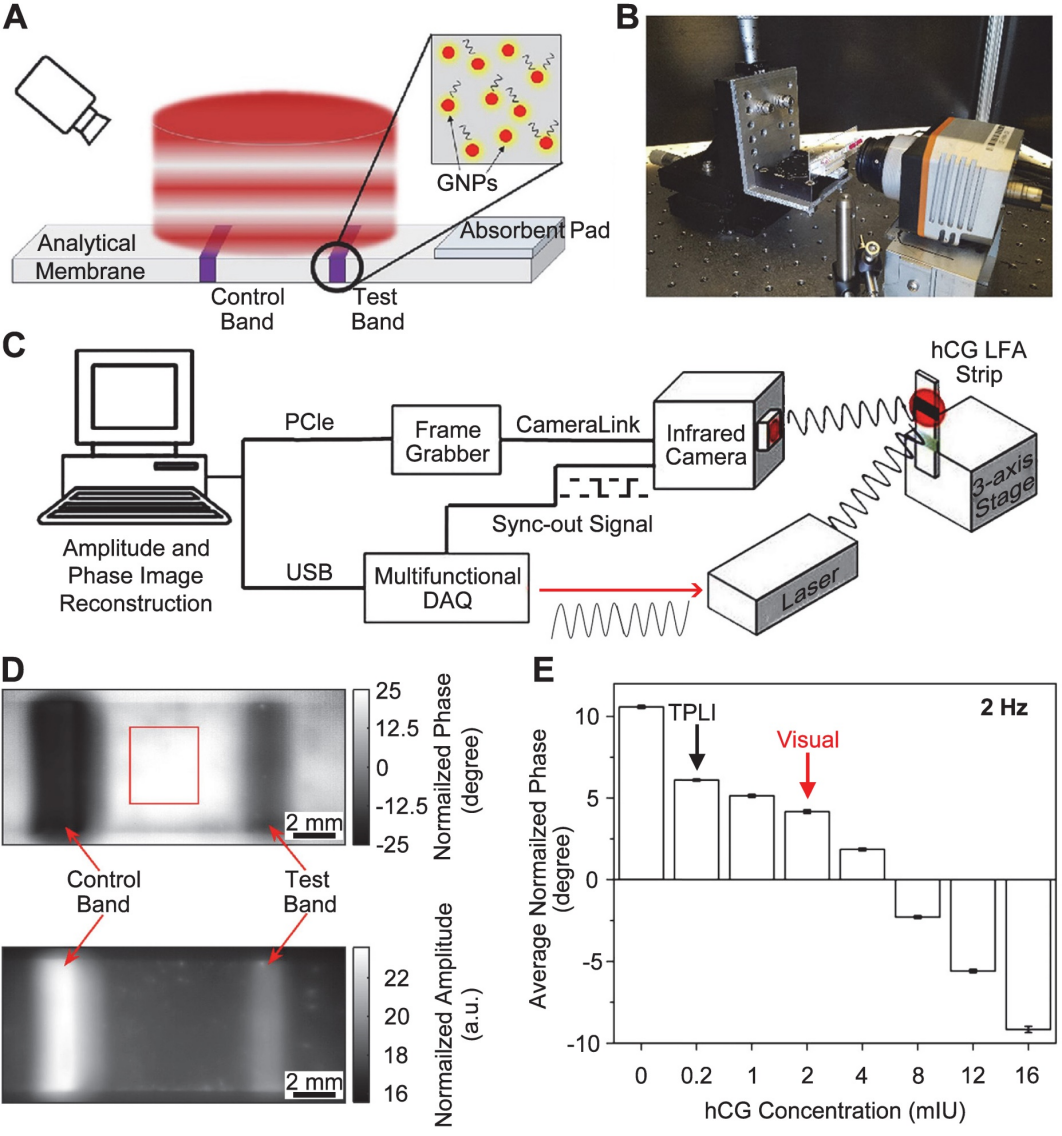
Thermophotonic lock-in imaging (TPLI) system for LFA. Schematics showing (A) the TPLI working principle and (B) a photograph of the experimental setup, and (C) the major components of TPLI system used for interpretation of LFA results. (D) TPLI phase (top) and amplitude (bottom) images of LFA strip obtained at hCG concentration of 16 mIU and 2 Hz modulation frequency. (E) Average normalized phase values at 2 Hz modulation frequency within the test band against different hCG concentrations. The arrows mark the detection threshold of visual and TPLI readouts. Adapted with permission from 71, copyright 2018 Elsevier.

When coupled with LFA, the TPLI provides a promising method utilizing thermal response of GNPs as the indicators. Interestingly, the TPLI is capable to inspect the depth of a feature by controlling the laser modulation frequency 68. This ability enables the detection of GNPs trapped on surface and inside the membrane. In a recent work, Ojaghi et al. developed a long-wave infrared TPLI system for the thermal interpretation of LFA strips 71. As shown in Figure 4A, the working scheme was to use a laser to first excite the region of interest (ROI) covering both control and test bands for the generation of thermal waves (i.e., Planck radiation), which was subsequently detected by an IR camera. Due to the spatial distribution of GNPs at various layers of the membrane, the diffusive thermal field was altered and resulted in a depth-integrated signal. Therefore, a phase shift was formed when the inner thermal wave reached the surface and contributed to the thermal radiation. Simultaneously, amplitude change of the radiometric signal was induced by the subsurface absorption of light that can also be used to quantify the concentration of GNPs and the analyte.

TPLI was then tested to enhance LFA performance. Figure 4B shows the setup for TP signal generation, where the testing strip was mounted on a stage to secure the position and height. A multifunctional data acquisition system was designed to generate analog reference and acquire data synchronously under different excitation cycles (Figure 4C). The as-obtained TP signal was then proceeded via a standard quadrature demodulation/ lock-in analysis to decipher amplitude and phase information. Typical TP phase and amplitude images of LFA strip were shown in Figure 4D at 2 Hz of laser frequency, where the control and test bands and the adjacent background (red box) can be resolved. Due to the different number of GNPs accumulated in those three regions, the corresponding thermal contrast could be observed in both images. Therefore, the variation of contrast in the test bands from different strips was used to correlate with the concentration of GNPs and analyte. Under the optimized modulation frequency (2 Hz), the average normalized phase signal decreases quantitatively as a function of hCG concentration (Figure 4E). Statistically, 0.2 mIU hCG in LFA could be detected through TPLI technique, while only 2 mIU hCG was reached by visual interpretation or optical reader. In addition to the sensitivity and quantification, large-field and simultaneous measurements of multiple LFA strips is possible and could be accomplished in a short time (i.e., 10 s at 2 Hz modulation frequency).

##### Reader development

The latest report of this technique was performed in a benchtop system (i.e., on an optical table, Figure 4B and C). The authors mentioned that the IR camera can be potentially replaced by cell-phone attachment IR cameras to reduce the cost and size of the reader 71. More information about reader development is not available.

##### Limitations

For further development of devices, the following constrains of current technique need to be addressed. Firstly, the instrumental cost needs to be reduced. In the present demonstration 71, IR camera, laser, multifunctional data acquisition board are required to collect the signal, and a computer is used to process the data. Secondly, the LOD improvement strongly depends on the laser modulation frequencies. Optimal modulation frequency that yields thermal diffusion length commensurate with the thickness of the LFA needs to be experimentally tested. This may pose a challenge when different types of LFA kits are tested.

#### Photothermal laser speckle imaging (PT-LSI)

##### Principle

Laser speckle imaging (LSI) is an interferometric technique to measure the optical contrast generated by the light scattering of a moving sample 72. When a laser illuminates a diffuse material, the highly coherent light produces granular interference pattern known as speckle 73. In a typical LSI, a digital camera records this laser speckle under very short exposure time (several milliseconds), recording the surface intensity pattern of light from a scattering medium. This intensity-fluctuation profile, through a simple Fourier transformation, enables statistical calculation and mapping of species dynamics 74. Collectively, many applications have been proposed and implemented in the fields of neuroscience, dermatology, and ophthalmology 75-77. Photothermal laser speckle imaging (PT-LSI) has advanced the conventional LSI with improved depth resolution by incorporating a pulse laser. This leads to the selective excitation of absorbers and allows for highly sensitive detection 78.

##### LFA demonstration

**Figure 5:**
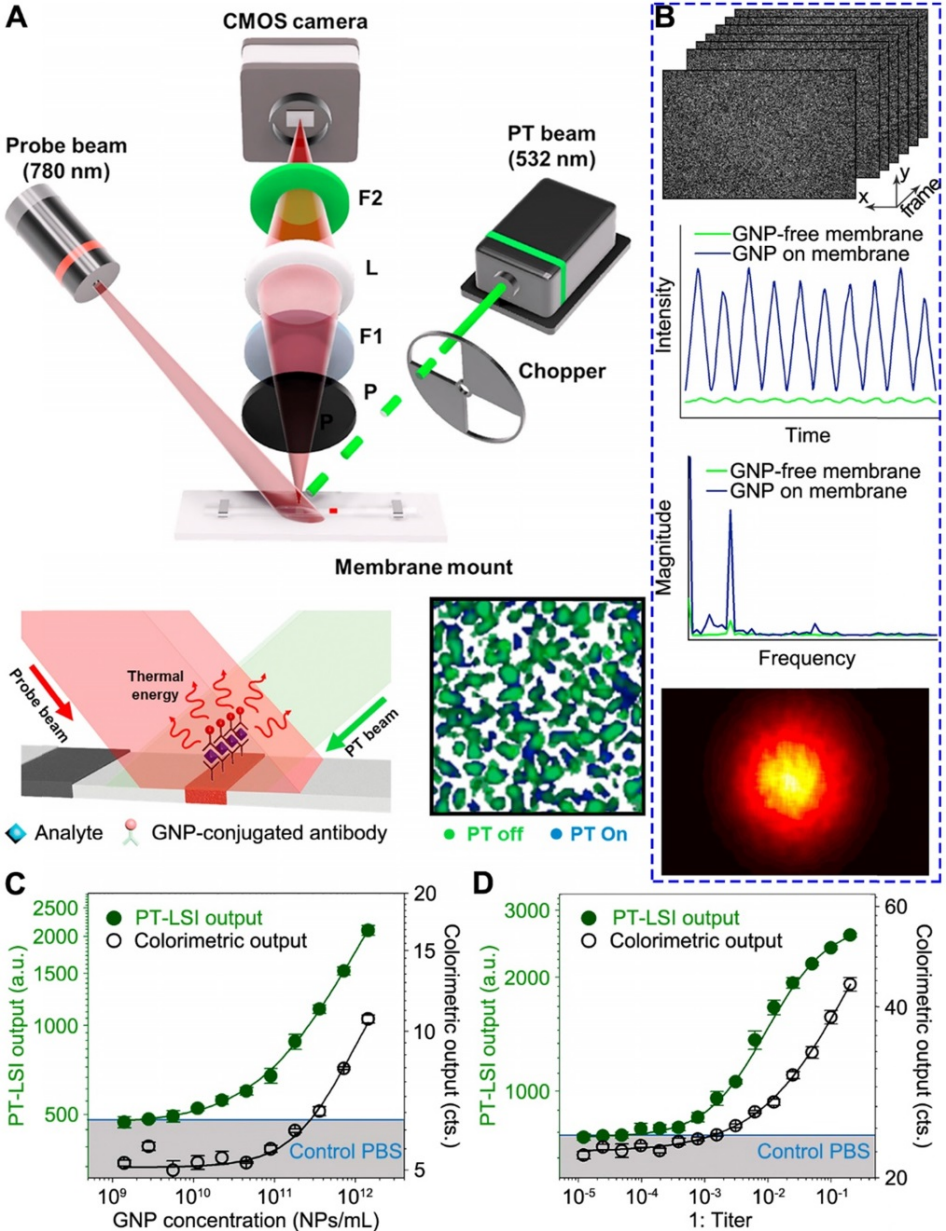
Photothermal laser speckle imaging (PT-LSI) system for LFA. (A) Schematics of the PT-LSI setup working on a mounted LFA strip. (B) Outline of the PT-LSI signal processing. The procedures include speckle images acquisition, pixel intensity fluctuation measurements, Fourier transformation of magnitude, and PT-LSI signal output. Dose response curves of PT-LSI signal of LFA for (C) GNPs and (D) CrAg detection. The blue line in both plots marks the noise-equivalent output, which was acquired with PBS buffer only. Adapted with permission from 79, copyright 2018 Elsevier.

Song et al. have recently demonstrated the use of PT-LSI for LFA 79. Due to the multiple layered structure of LFA strips, the probe light scatters randomly and forms speckle patterns. The working principle, as shown in Figure 5A, takes advantages of a PT laser with 532 nm beam to specifically excite the GNPs on membrane. As a consequence, the GNPs generate heat and change the speckle pattern of LFA membrane by the scattered light of 780 nm probe laser. Instead of using IR camera as the sensor, the speckle pattern change was monitored by an optical sensor (i.e., CMOS camera), which reduces cost compared with IR cameras. An optical chopper is employed to modulate the intensity of PT light to minimize external noise, such as laser intensity variation. The excited GNPs generate heat that alters the refractive index and causes thermal deformation of the membrane, resulting in the speckle pattern change. This change is associated with the number of GNPs reside, which can be used to quantify the target concentration. A custom-built program implemented in MATLAB was designed to process the PT-LSI signal. The analytical procedure is outlined in Figure 5B (top to bottom). Firstly, the pixel intensity of each PT-LSI image obtained at different time periods is characterized by a fluctuation at the same frequency of the PT light modulation. Note, the presence of GNPs gives a much stronger signal than the background signal from LFA membrane. Secondly, the intensity fluctuation profile is Fourier transformed into magnitude as a function of frequency, which is further mapped into a PT-LSI image based on the corresponding pixels. Lastly, the averaged PT-LSI signals, referred to as “PT-LSI output” over the ROI (i.e., control and test bands), quantify the concentration of GNPs and analytes. As evidenced in Figure 5C and D, the PT-LSI result showed enhanced analytical performance over the colorimetric signal in quantifying the concentration of GNPs solution and CrAg sample. The LOD of the PT-LSI coupled LFA was 125- fold and 68-fold lower than that of the colorimetric LFA for detecting GNP and CrAg, respectively.

##### Reader development

As mentioned by the authors, the PT-LSI reader can be readily miniaturized to a portable size with inexpensive consumer-grade components 79. For instance, the optical chopper for PT light modulation can be replaced by diode laser current modulation with a microcontroller, and the high-speed CMOS camera can be substituted with webcams that is less than $20. In addition, the PT light source can be a laser pointer. Alternatives such as using high-speed field programmable gate array devices and pulsed or coded PT light illumination were also proposed to increase the data analysis speed over the current results (i.e., 2.5 min).

##### Limitations

The robustness of using laser pointers and web camera as the key components for portable reader should be rigorously tested. Especially, the power stability of laser pointer will be a major obstacle for consistent performance during long term usage. In addition, the data processing for speckle images of PT-LSI technique should also be considered for resource-limited settings.

### Photoacoustic (PA) enhanced signal

#### Principle

Photoacoustic (PA) effect is another interesting phenomenon induced by the laser absorption of chromophores and pressure wave generation 80. Detection method using PA signal was first developed in the 1970s 81. Essentially, the PA technique provides quantitative results based on three steps, including the optical absorption, the energy conversion from light to heat, and the thermal expansion of the adjacent media that produces acoustic waves as PA signal 82. As a result, the PA signal increases with the absorbed energy and indicates the optical absorption of a sample. In addition, the PA imaging has deeper penetration due to the ultrasound transmission and sensitive detection, compared with limited light penetration in optical imaging 83. Taken together, the PA technique has been very attractive to the analytical community and its integration with current diagnostic platforms (e.g., ELISA) 82 is promising to move from the laboratory research to preclinical and clinical realities 83, 84.

#### LFA demonstration

**Figure 6:**
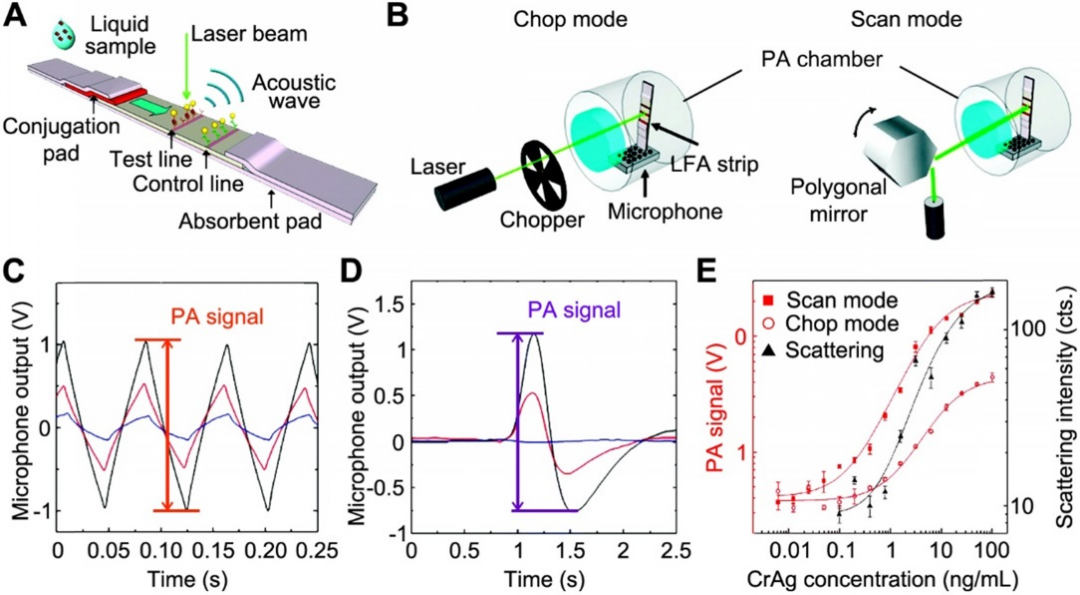
Schematics of the (A) working principle of PA-LFA; and (B) detection modes of PA-LFA. Note an air-tight chamber is used to block the environmental variations and the microphone is for the PA signal amplification. The measured PA waveforms with different number of GNPs absorbed on the strip in chop mode (C) and scan mode (D), respectively. For both plots, the peak-to-peak value was marked as the PA signal; and the color curves mark the concentration of GNPs applied: 10^11^ mL^-1^ for black, 3×10^10^ mL^-1^ for red, and 0 for blue (control). (E) Comparison between diagnostic performance of PA measurements under the scan mode and chop mode and colorimetric result. Adapted with permission from 85, copyright 2016 The Royal Society of Chemistry.

Zhao et al. first explored the combination of PA technique with LFA 85. Figure 6A shows that, when illuminated by a CW laser beam, the GNPs captured within test/control bands generate PA signal due to the thermal expansion of the adjacent air. Meanwhile, the different density of GNPs at the test band leads to the amplitude change of PA signal, which indicates the analyte concentration. To ensure the precise and quantitative measurements, the LFA strip was enclosed in a small-volume and air-tight chamber with minimized vibration from the environment and the strip itself (see Figure 6B). Also, two detection modes were developed, where the “chop mode” employed an optical chopper to modulate the laser intensity and the “scan mode” used a rotating polygonal mirror for the scanning across the ROI. Notably, the peak-to-peak value of the signals (Figure 6C and D) was marked as the PA signal amplitude and further used to calculate the analyte concentration. Due to the adsorption of light by the strip itself, a strong background signal was observed in the “chop mode” (blue trace in Figure 6C), while in the “scan mode”, the background signal was reduced (blue trace in Figure 6D). This is because, without chopping the laser, the LFA strip absorbs a constant amount of the excitation light during the scan and the laser stimulation generates a GNP-specific signal when scanning across the test band. Using CrAg LFA kits as an example, PA method could significantly enhance the sensitivity over the colorimetric detection. Figure 6E shows the dose-response curves of the colorimetric analysis, chop mode, and scan mode of PA detections, giving LOD of 1.1 ng·mL^-1^, 0.57 ng·mL^-1^, and 0.010 ng·mL^-1^, respectively. These results clearly demonstrated that the PA technique offers a powerful means to amplify the signal, enabling a more sensitive (110-fold enhancement) and quantitative analysis for LFA.

#### Reader development

The authors proposed several options to miniaturize the size and reduce the cost of PA detection system 85. For instance, it is technically available to replace laser with a LED that matches the absorption of the GNPs and integrate the light source, microphone, the detection chamber, and readout circuit into a palm-size box. Recently, Zhang et al. proposed a miniaturized PA cell for testing of blood glucose on paper-based devise 86. The Teflon-made PA cell is a cubic structure with 40 × 20 × 20 mm in dimension, where a sample holder, a cylinder chamber, and a microphone are configurated together inside for signal generation and acquisition. The small size of the chamber (~0.2 cm^3^) prevents the sound loss during the testing. The whole cell is sealed to enable a stable environment for minimal fluctuation of signals. Future work may extend to other paper-based assays (e.g., GNPs-based LFA).

#### Limitations

In the work by Zhao et al., the LFAs were dried for 15 min before tested by PA method 85. However, it is noted in most commercially available LFA kits, the results should be read when the strip is wet for more accurate interpretations. It is important to validate this technique for wet LFA readings since low percentage of the acoustic wave is able to travel through water-air interface. Again, reproducibility issue raises the users' concerns due to the intrinsic point-scanning reading of PA technique.

## Summary and Outlook

In this article, we have highlighted the laser- induced signal amplification techniques on GNPs- based LFA reported in the past decade. GNPs can be excited by the laser and convert the absorbed energy into enhanced electromagnetic field or heat due to the LSPR. Such responses of GNPs provide new sensing modes that have been successfully integrated on LFA, including SERS, thermal contrast, thermophotonic, speckle, and photoacoustic signals. Different reader systems including hardware (e.g., laser source and detector) and software (e.g., signal acquisition and data proceeding) have been introduced for each technique implementation. Compared with the colorimetric readouts of LFA, those techniques show improved analytical performance upon the sensing of analytes. Promising as they are in this technological development, we are expecting to witness more competing products in the coming decade.

These signal amplification strategies are more straightforward than the colorimetric enhancement strategies since the latter one requires specifically designed detection agents (e.g., enzymes coated GNPs) or additional reagents for improved signal readouts. As clearly illustrated from the above- mentioned examples, the laser-assisted techniques detect the signal from a completed strip without extra assay operation and can be readily implemented on commercially available LFA kits. However, there are several limitations: 1) for SERS-LFA, it suffers from the high assay expenses and special SERS tags preparation; 2) for the photothermal-related detection, the bulky instrumentation needs to be further improved with lower-cost components; 3) clinical assessment is also highly demanded to evaluate the enhanced sensitivity versus the likelihood of false-positives for all techniques; and 4) the point-focus scanning (e.g., TCA and PA methods) needs to be optimized for rapid and robust readouts. The comparison of each method viewed from other aspects has been summarized in Table 1.

With these developments for LFA signal enhancement, we envision that diagnostic performance of an assay is one of several critical parameters towards practical applications. Particularly, minimizing non-specific binding and increasing specific binding at test bands of LFA strips are the key to reduce background signals and interpret accurate results at low target concentrations (vs. false positives). Other factors such as the optimization of whole detection system, including signal generation, miniaturization, cost, operation time, automation, and data analysis, should also be considered from the POC perspective 87. Toward this end, the development of devices may be divided into three stages: 1) initiate research study to demonstrate the feasibility of integrating new detection techniques on LFA strips and the research outcomes could serve as a guideline to develop portable systems; 2) prototype a benchtop system consisting of lower-cost and smaller components, which offers on-demand tests and can be installed in hospitals or central referral laboratories; and 3) develop a handheld system that can be used in a wide range of environmental settings and fulfill the need of on-site decision making with high sensitivity. Such device development is an important step to translate the innovation from benchtop to bedside 88. Licensing techniques from academic institutions to startup companies or incubators can further translate these findings into the clinics yet remains another challenge (i.e., “valley of death” 89). As a good reference, the development of fluorescence- based LFA directs a pathway for the researchers who are interested to pursue further clinical translation, such as LFA manufacturing, fluorescence label design, and instrument optimization. For example, the deployment of upconverting NPs in LFA reduces the background signal from the strip 90; and there are many commercially available fluorescence readers in the market.

For future POC diagnostics, the highly flexible formats of LFA allow further integration of novel materials and techniques. For instance, GNPs with varied shapes and structures provide a simple yet efficient way for the sensitivity and accuracy improvement of the LFA, while it's now feasible to prepare them in a controlled manner 91, 92. It is promising to apply them into LFA for new signal-transducer principles and substantial signal enhancement. While for the size-dependent signal transduction, its response usually increases with the GNPs size. However, the membrane structure of LFA strips traps the large particles, and it leaves the assay incomplete. Therefore, rather than enlarging the size of GNPs for stronger signals, the priority should be put in choosing NPs with proper size without difficulties in completing the assay. Also, the multiplexing of LFAs is a critical area for the development of the POC diagnostics. In this case, the creation of pads or other platforms and efficient conjugation events will be of great importance 3, 93, 94. Microfluidic platforms that are able to precisely manipulate the flow rate of sample and the migration of probes and targets allow for high-throughput screening applications. There will be some potential opportunities to combine the advantage of these two platforms 95, 96. Smartphone has been developed as a platform for POC diagnostics in recent years, offering great opportunities for delivering healthcare to resource-limited settings due to its availability 97. The integrated components (e.g., camera, chips, and add-on thermal camera from FLIR^®^) of smartphone are extremely suitable for signal enhancement and detection of LFA 98-100. While not a focus in this paper, enhancing the optical signal of GNPs in LFA by increasing the probe density around the analytes like self-assembly and aggregation has been well documented in recent papers 101-103. Ultimately, we hope that this article can be a valuable source to the communities in academia and industry for future work in this important and emerging field.

## Figures and Tables

**Table 1:** Comparison of the presented signal amplification techniques and detection methods.

Enhancement strategy	Surface enhanced Raman scattering (SERS)	Thermal contrast amplification (TCA)	Thermophotonic lock-in imaging (TPLI)	Photothermal laser speckle imaging (PT-LSI)	Photoacoustic (PA)
**Probes used**	SERS tags of 45 nm GNPs and 85 nm Au-Ag NPs	30-100 nm GNPs	30 nm GNPs	30 nm GNPs	30 nm GNPs
**Detection system**	CW laser Raman spectroscopy	CW laser IR camera	CW laser IR camera	CW laser CMOS camera	CW laser PA detector
**LOD enhancement^a^**	10^4^-fold and 4-fold	8~256-fold	10-fold	68-fold	110-fold
**Multiplexing**	Yes	N/A			
**Reading algorithm**	Point or line scanning	Point scanning	LFA area imaging	LFA area imaging	Point scanning
**Automation^b^**	No	Yes	Yes	Yes	No
**Data acquisition time**	6 min and 5 s	10 min	10 s	20 s	~5 s^c^
**Clinical cohort study**	No	Yes	No	No	No
**Current status**	Laboratory R&D	Startup company^d^	Laboratory R&D	Laboratory R&D	Laboratory R&D
**Ref**	37, 54	61, 63, 66, 67	71	79	85

a: Comparison of LFA LOD with colorimetric readouts; b: Refer to the data acquisition and analysis; c: Estimated by the scanning speed of 3 mm/s; d: Vigilant Diagnostics

## Abbreviations

Ag: silver
Au: gold
Pt: platinum
SiO_2_: silica
ELISA: enzyme-linked immunosorbent assay
FDA: Food and Drug Administration
CMOS: complementary metal-oxide semiconductor
LED: light-emitting diode
